# Research on Downhole MTATBOT Positioning and Autonomous Driving Strategies Based on Odometer-Assisted Inertial Measurement

**DOI:** 10.3390/s24247935

**Published:** 2024-12-12

**Authors:** Mingrui Hao, Xiaoming Yuan, Jie Ren, Yueqi Bi, Xiaodong Ji, Sihai Zhao, Miao Wu, Yang Shen

**Affiliations:** 1 Campus School of Mechanical and Electrical Engineering, China University of Mining and Technology Beijing, Beijing 100083, China; haomingrui@163.com (M.H.); zsh@cumtb.edu.cn (S.Z.);; 2 China Coal Technology & Engineering Group Taiyuan Research Institute Co., Ltd., Taiyuan 030006, China; emyxm@126.com (X.Y.); yueqibi@outlook.com (Y.B.); 3 Department of Mechanical Engineering, Taiyuan Institute of Technology, Taiyuan 030008, China; renji5277@163.com; 4 State Key Laboratory of Mechanical Behavior and System Safety of Traffic Engineering Structures, Shijiazhuang Tiedao University, Shijiazhuang 050031, China; 5 School of Vehicle and Transportation Engineering, Tsinghua University, Beijing 100083, China; shenyang@mail.tsinghua.edu.cn

**Keywords:** underground coal mine, auxiliary transportation, MTATBOT, SLAM, explosion-proof, autonomous driving

## Abstract

In response to the current situation of backward automation levels, heavy labor intensities, and high accident rates in the underground coal mine auxiliary transportation system, the mining trackless auxiliary transportation robot (MTATBOT) is presented in this paper. The MTATBOT is specially designed for long-range, space-constrained, and explosion-proof underground coal mine environments. With an onboard perception and autopilot system, the MTATBOT can perform automated and unmanned subterranean material transportation. This paper proposes an integrated odometry-based method to improve position estimation and mitigate location ambiguities for simultaneous localization and mapping (SLAM) in large-scale, GNSS-denied, and perceptually degraded subterranean transport roadway scenarios. Additionally, this paper analyzes the robot dynamic model and presents a nonlinear control strategy for the robot to autonomously track a planned trajectory based on the path-following error dynamic model. Finally, the proposed algorithm and control strategy are tested and validated both in a virtual transport roadway environment and in an active underground coal mine. The test results indicate that the proposed algorithm can obtain more accurate and robust robot odometry and better large-scale underground roadway mapping results compared with other SLAM solutions.

## 1. Introduction

Coal mine intellectualization is the core technical support for the high-quality development of the coal industry. Particularly for underground coal mines, the harsh working conditions, heavy labor intensities, and frequent mining accidents make it increasingly difficult for enterprises to recruit employees engaged in subterranean environment works. Therefore, the construction of production automation, equipment robotization, and unmanned operation based on the application of artificial intelligence, robot technology, industrial Internet, cloud platforms, and other labor-saving technologies throughout the entire underground mining process has become the main development goal of the coal industry for the future [1,2]. Compared to the main transportation system, which is responsible for coal transportation, the auxiliary transportation system in underground coal mines is in charge of the transport of production consumables, working equipment, and subterranean personnel. Because of the diverse transport objects, complex driving environments, and heavy transport tasks, the auxiliary transportation system has become one of the most labor-intensive and accident-prone parts of the whole underground mining process. Therefore, an intelligent and unmanned auxiliary transportation system can effectively reduce the amount of subterranean labor, increase mining efficiency, and lower mining accident rates. Several coal production groups have proposed construction objectives for intelligent auxiliary transportation, aiming to develop a model that incorporates “path planning” and “unmanned driving” technologies [3].

However, the current auxiliary transportation system in underground coal mines mainly uses manually driven vehicles to perform transport tasks. Depending on different transportation conditions, auxiliary transport equipment can be broadly classified into two categories: railed locomotives and trackless vehicles. Compared with railed locomotives, trackless auxiliary vehicles can perform transport tasks from point to point, avoiding the trans-shipment process, which is more efficient, flexible, and labor-saving. Trackless auxiliary transportation can reduce the proportion of people engaged in transport operations underground from more than one-third of rail transportation to about one-tenth, effectively reducing the labor intensity of workers.

Currently, trackless auxiliary transportation mainly depends on explosion-proof rubber-wheeled vehicles. However, these vehicles are mainly driven by explosion-proof diesel engines, which depend on hydraulic or mechanical transmission and rely on human operation, making it hard to achieve automatic control and unmanned transportation. The existing trackless auxiliary transport system adopts different types of explosion-proof rubber-wheeled vehicles for transporting diverse objects, resulting in a wide variety of transport equipment and heavy maintenance work. These vehicles have common problems such as a low intactness rate and a high failure rate. Personal injuries and underground equipment damage accidents caused by such vehicles are common, sometimes causing coal mine fire accidents and resulting in serious casualties and property losses. In 2011, employees of the Hanglaiwan Coal Mine belonging to Shaanxi Nonferrous Yulin Coal Industry Co., Ltd. took a rubber-wheeled trackless vehicle into the auxiliary inclined shaft for 200 m and found that the vehicle could not shift gears normally and that the brakes had failed. The vehicle rushed into the left side of the tunnel during the sliding process, killing four people. In 2013, Shanxi Liliu Xinrui Coal Industry Co., Ltd. had a transportation accident due to the brake failure of a rubber-wheeled trackless vehicle, which rear-ended the equipment in front, resulting in six deaths and direct economic losses of CNY 5.91 million.

In short, relying on the existing equipment, it is difficult to support the standardization and automation of underground transportation. Therefore, the use of autonomous driving and robot technology to upgrade and iterate the auxiliary transportation of coal mines can effectively reduce accidents and ensure production safety, which is of great significance. In response to the above problems, in this paper, a mining trackless auxiliary transportation robot (MTATBOT) used for material and consumable distribution in the underground coal mine is introduced, powered by explosion-proof lithium batteries and equipped with an explosion-proof wired control system. The robot can be controlled by a remote operation platform or an onboard autopilot system to execute unmanned underground transportation. This paper describes the system composition and functional implementation of MTATBOT, aiming to provide an intelligent transport terminal and feasible solution for intelligent auxiliary transportation in underground coal mines.

The remainder of the paper is organized as follows. Section 2 presents an overview of the related unmanned technology and equipment development of intelligent mining, the general structure of the MTATBOT is shown in Section 3, and Section 4 describes the autopilot system design of the robot. The robot’s dynamic model and control strategy are presented in Section 5. In Section 6, the simulation test and field experiment results of the robot virtual model are presented. This paper ends with a conclusion in Section 7.

## 2. Prior Works and Technical Challenges

To enhance transportation efficiency and ensure operational safety, coal mine designs have dedicated enclosed areas and tunnels for autonomous vehicles, thereby minimizing interference from unpredictable factors, such as pedestrians. In recent years, autonomous driving has made great progress in some mining and machinery companies, especially in some open-pit mines. The productivity benefits of autonomous open-pit mining truck fleets have helped to reduce costs by around 20 percent, and the autonomous trucks effectively shield employees from dangerous situations [2]. In the open-pit mining area, benefiting from the advantages of the global navigation satellite system (GNSS), the autonomous driving system can more easily obtain accurate location information from mining trucks, which is the premise and basis of automatic and unmanned transportation. In addition, the transport objects of open-pit mines are relatively fixed, mainly focusing on coal or other ores, which makes it easier to achieve standardized and automated transport processes. However, considering that GNSS signals cannot spread in a subterranean environment, the precise localization of autonomous driving vehicles in underground terrains must be solved with other solutions. Additionally, the special features of underground coal mine environments pose challenges to autonomous driving vehicles, including limited lines-of-sight, large variations in illumination, obscurants (e.g., dust, fog, and moisture), restricted working spaces, similar long-narrow roadways, degraded perception and sensing, constrained communication, radio frequency propagation challenges, explosion-proof requirements of electric components, and increasingly complicated environment with mining operations. However, the most challenging of all is the combination of all of the above features.

Thus, researchers have performed much work to promote the application of relative technologies. Dong et al. proposed analytical and iterative velocity-free localization methods in complex and dynamic mining conditions [4]. By leveraging active sources, laser rangefinders, and proximity sensors, along with localization techniques, the precise localization of autonomous rock drilling jumbos and explosive charging vehicles in deep underground mines was achieved. However, this method depends on the pre-deployment of a sensor network, leading to increased economic costs and infrastructure investment, particularly in large-scale underground coal mine roadways. Ebadi et al. introduced a comprehensive autonomous mapping and positioning approach for navigating perceptually challenging subterranean environments based on simultaneous localization and mapping (SLAM) technology [5,6,7]. Depending on the centralized multi-robot SLAM system, a robust estimate of the trajectories of multiple robots in large-scale, unknown, and complex subterranean environments can be obtained. Another algorithm, named range-aided pose graph-based SLAM, was introduced by Funabiki et al. to execute position estimates in subterranean perceptually degraded environments with the help of sparsely deployed ranging beacons [8]. Kim et al. developed an autonomous driving robot that drives and returns along a planned route in an underground mine tunnel through a machine vision-based road sign recognition algorithm [9]. Stefaniak et al. integrated the inertial measurement unit (IMU) and dynamic time warping (DTW) algorithms to locate the underground mine LHD and obtained robust performance [10,11]. A search-and-rescue robot system with explosion-proof and waterproof functions used for remote sensing of the underground coal mine environment was introduced by Zhao et al. [12]. In addition, ultra-wide-band UWB technology is increasingly being used to locate personnel and equipment in underground coal mines [13], and it is also considered to be helpful for underground driverless vehicles. However, limited by the features of the underground coal mine roadway, the construction of a UWB positioning network covering the entire auxiliary transportation roadway is hard to implement and incurs high costs. In addition, considering the relatively high speeds and safety requirements of underground transport vehicles, the dynamic positioning accuracy and signal stability of UWB technology in an underground environment need to be further tested and improved. Briefly, the above state-of-the-art solutions provide useful and positive references for intelligent unmanned auxiliary transportation in underground coal mines.

**Figure 1:**
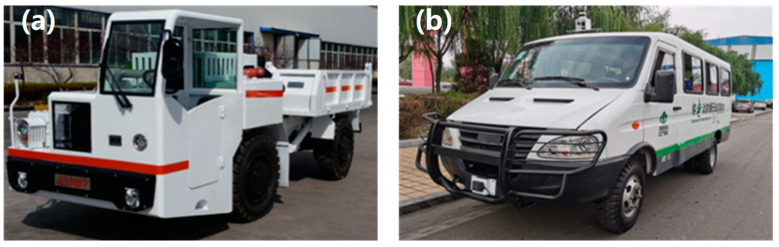
Explosion-proof rubber-wheeled vehicles manufactured by China Coal Technology & Engineering Group, Taiyuan Research Institute. (**a**) A traditional explosion-proof rubber-wheeled vehicle for material transport; (**b**) an autonomous driving prototype of an underground coal mine transport vehicle.

Moreover, in recent years, several kinds of autonomous driving technology validation models of underground coal mine transport vehicles have been developed by equipment manufacturers and research institutions, as shown in Figure 1. These models are mainly converted from the existing explosion-proof trackless rubber-wheeled vehicles by adding environmental sensors, such as Lidar, cameras, and millimeter wave radar. Some autonomous driving algorithms for underground coal mine environments were developed and tested based on these prototypes. However, the automatic driving modification of existing explosion-proof trackless vehicles cannot meet the standardized, continuous, and unmanned requirements of intelligent auxiliary transport systems.

The MTATBOT introduced in this paper is a new type of robotized coal mine auxiliary transport system, which is specially designed for unmanned material distribution in underground coal mines. Compared with other underground unmanned driving solutions that transform existing vehicles into wire-controlled models through after-installation modification, the MTATBOT takes into account the particularity of the underground environment of coal mines and the wire-controlled requirements of autonomous driving at the beginning of its design. It adopts explosion-proof wire-controlled technology to achieve high-precision electronic control of chassis movements, meeting the control requirements of the autonomous driving system. Meanwhile, the environmental perception system is pre-deployed according to the appearance and structural characteristics of the robot to achieve detection and perception without blind spots, meeting the strict safety requirements when operating in the limited space of underground roadways. In the following article, the structural composition and functional design of the robot system will be described in detail.

## 3. General Description of the MTATBOT

The application of the proposed MTATBOT system focuses on the unmanned transportation of materials and consumables in large-scale underground coal mines. The robot is designed to reduce reliance on human transport personnel, enhance transportation efficiency, and ensure operational safety. To adapt to the complex environments of large mines, the robot must possess a long battery life, be compact, have off-road capabilities, and possess a certain level of autonomous decision-making ability. Equipped with built-in environmental sensing and autonomous decision-making systems, the robot can plan optimal routes to achieve efficient, energy-saving, and point-to-point transportation in an underground environment. Moreover, given the spatial constraints of underground tunnels, varying transportation requirements, and the need for safe obstacle avoidance during transport, the system focuses on multi-sensor fusion to enhance the accuracy of perception. To boost its autonomous decision-making capabilities, advanced artificial intelligence algorithms are integrated into the system, enabling it to respond quickly in dynamically changing environments to avoid potential obstacles and hazards.

**Figure 2:**
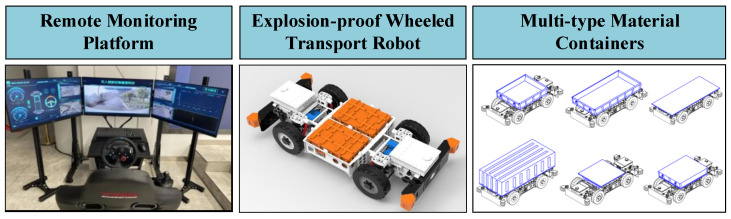
The trackless auxiliary transportation robot system used for material distribution in underground coal mines.

Additionally, the robotic system features a modular design, making maintenance and functionality expansion convenient to meet various operational needs and scenarios. This innovation not only advances intelligent mine transportation but also lays a solid foundation for the development of smart mines in the future. With a modular structural design, the MTATBOT breaks through the conventional structure of explosion-proof trackless vehicles and divides transport operations into different functional units. For different transport objects, the required functional units can be combined into a suitable transport vehicle, thus reducing the variety of vehicle types and facilitating standardized transportation. As shown in Figure 2, the MTATBOT consists of three main parts, including a remote monitoring platform, an explosion-proof wheeled transport robot, and multi-type material containers.

### 3.1. Remote Monitoring Platform

The remote monitoring platform is the command and dispatch center of the MTATBOT. The platform can be independently arranged in the ground control center or integrated into the comprehensive dispatching system of the coal mine. The remote monitoring platform has three main functions. First, it is in charge of processing task information on required material types, underground destinations, and the locations of available wheeled transport robots. The robots that can perform the transport task will be identified based on this information. Secondly, the remote monitoring platform is also responsible for calculating the global planning path of the robot from its current location to the required destination based on real-time underground traffic conditions. The generated global planning path is then released to the identified robot as the task navigation information via the wireless communication network of the coal mine. Third, the platform can monitor the robot’s operational status and execute remote takeover as needed to ensure transportation safety.

### 3.2. Explosion-Proof Wheeled Transport Robot

**Figure 3:**
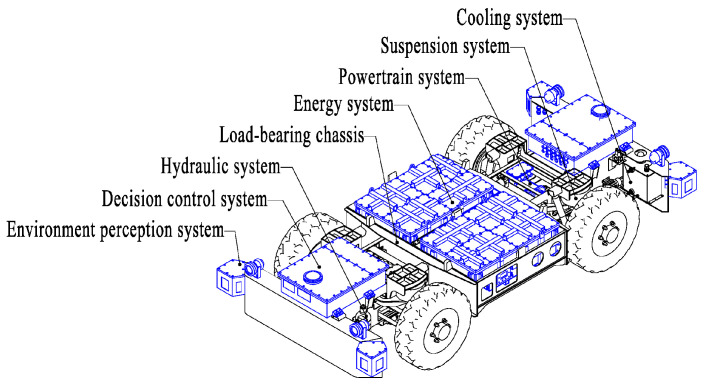
Structural configuration of the explosion-proof wheeled transport robot.

An explosion-proof wheeled transport robot (EWTBOT) used as an automated guided vehicle is the transport actuator of the MTATBOT. The EWTBOT is designed for the complex operating environment and special working conditions of underground coal mines and is equipped with an autopilot system as its control center and an explosion-proof wheeled electric chassis as its walking device. The configuration structure and chief components of the explosion-proof wheeled electric chassis are shown in Figure 3. The chassis is designed with a front-to-back symmetrical structure to achieve good maneuverability and bi-directional travel capability. Meanwhile, considering the limited underground working space, the chassis adopts a low-profile design with dimensions of a length of 4500 mm, a width of 2000 mm, and a height of 1000 mm. The chassis is powered by batteries and is zero-emission. Two blocks of explosion-proof lithium batteries are mounted in the middle of the chassis, providing 64 kWh of energy. The onboard energy system gives the robot a maximum driving range of 80 km to meet the demand of at least one round trip of material transportation in a large-scale underground coal mine. Furthermore, in order to solve the problems of driving range anxiety and excessive charging time faced by current explosion-proof electric vehicles, the chassis has a quick-change battery function that allows battery packs to be replaced in ten minutes. In addition, the robot is equipped with a four-wheeled independent suspension system to achieve good driving stability when traveling in complicated underground road conditions. The hydraulic system of the chassis is powered by an explosion-proof oil pump motor to perform the braking function of the robot.

**Figure 4:**
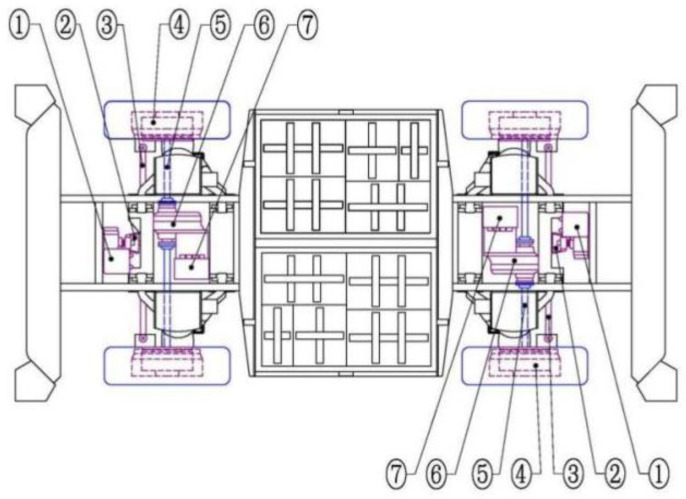
Powertrain arrangement of the explosion-proof wheeled transport robot. ① Explosion-proof electric steering gear; ② Steering swing arm; ③ Steering tie rod; ④ Wheel-side enclosed wet brake; ⑤ Drive axle shaft; ⑥ Reducer and differential gear; ⑦ Explosion-proof permanent magnet motor.

The autopilot system of the EWTBOT includes an environment perception system and a decision control system. Two explosion-proof electric control boxes are symmetrically placed at the front and rear ends of the chassis to host onboard electric components and computing units. Four explosion-proof electric control boxes are arranged separately at the corners of the chassis, in which the sensors required for the environment perception system are placed. The powertrain system arrangement of the EWTBOT is shown in Figure 4. The robot is equipped with two sets of driving units, each consisting of a 46 kW explosion-proof permanent magnet motor and a reducer with differential function. The power system enables the robot to reach a maximum travel speed of 40 km/h and a maximum climbing capacity of 14°. Meanwhile, in order to obtain efficient maneuvering in limited spaces underground, the robot is equipped with two sets of Ackerman steering mechanisms to enable four-wheel steering and ensure a small turning radius. Moreover, each tire of the robot is equipped with a wheel-side enclosed wet brake. Considering the safety of braking, the brake adopts a safety-type operating mode that is spring-applied and hydraulically released, which will be automatically locked when the robot loses power. In addition, the robot is equipped with polyurethane-filled tires, increasing its adaptability to complex subterranean road conditions. The main parameters of the EWTBOT are listed in Table 1.

### 3.3. Multi-Type Material Containers

**Figure 5:**
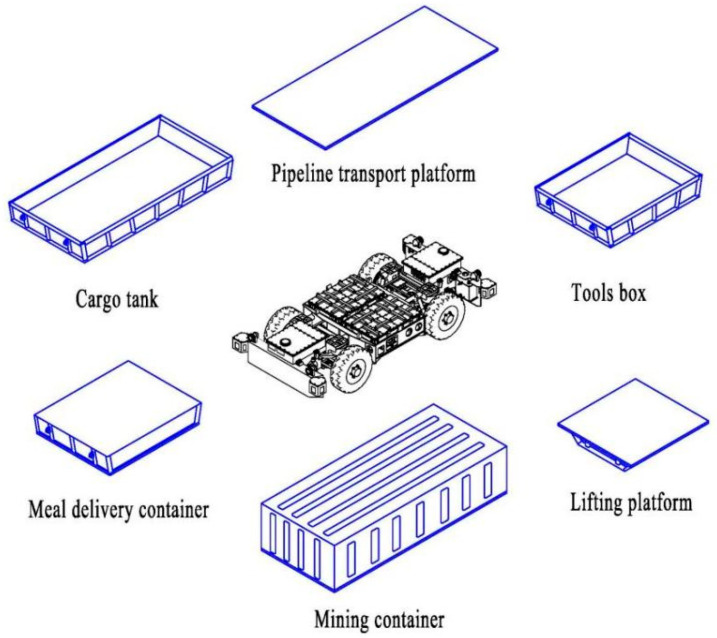
Multi-type material containers of the trackless auxiliary transportation robot system.

Muti-type material containers are a series of removable top-loading devices that could be quickly changed and installed on the EWTBOT. As shown in Figure 5, several kinds of material containers are designed to meet the transportation needs of various materials during underground production in coal mines, such as anchor rods, anchor cables, building materials, pipes, meals, and other spare parts or consumables. The volume and shape of the containers can be customized according to different mines’ demands. All kinds of loading containers are equipped with a unified installation interface and are easy to install on the robot. After receiving the transportation order from the remote monitoring platform, the EWTBOT selects a suitable container according to the task requirements and then executes the material delivery task. Therefore, the robot system is conducive to the standardization and centralization of coal mine materials management and the effective reduction of transport vehicle types. Furthermore, the material containers can be replaced with other operating agencies, such as robotic arms, lifting platforms, and cable reels, to convert the robot into a mobile operating platform.

## 4. Autopilot System of the MTATBOT

### 4.1. Autopilot System Configuration

**Figure 6:**
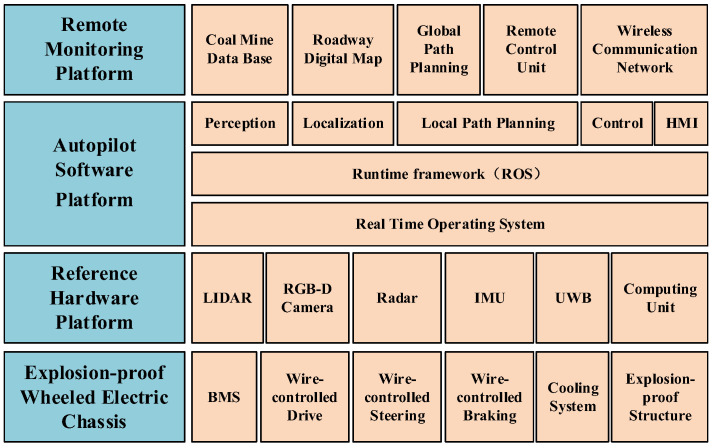
Autopilot system configuration of the trackless auxiliary transportation robot system.

The MTATBOT has two working modes: a remote control mode and an autopilot mode. When it is in the autopilot mode, the robot can automatically perform underground transportation. The autopilot system configuration of the MTATBOT is shown in Figure 6, including the remote monitoring platform, the autopilot software platform, the reference hardware platform, and the explosion-proof wheeled electric chassis. Except for the remote monitoring platform, all other systems are arranged on the EWTBOT. The reference hardware platform mainly consists of sensing devices, positioning modules, and a computing unit. The autopilot software platform can be further divided into three parts: a real-time operating system, a runtime framework, and functional modules. The real-time operating system adopts an Ubuntu-embedded operating system based on a Linux core, using the interaction interface between the software system and the hardware platform. The runtime framework adopts a robot operating system (ROS) that can provide a complete development toolkit, a flexible computing scheduling model, and rich debugging tools. The functional modules include a series of software packages that are mainly used to implement application-level algorithms and procedures for autopilot functions, such as perception, localization, path planning, and motion control. The explosion-proof wheeled electric chassis is the actuator of the autopilot system, which adopts explosion-proof wire control technology to realize the horizontal and vertical motion of the robot.

Compared to the variable ground transportation environment, the transport path of underground coal mines is relatively fixed. When the MTATBOT receives the transport task and destination information, its remote monitoring platform will calculate the global planning path based on the information on current underground traffic conditions and the available EWTBOT’s location. After receiving the start command, the identified EWTBOT will perform the transport task along the global planning path. At the same time, considering that the underground coal mine roadway is an environment shared by equipment and personnel, the actual trajectory of the robot must take into account information regarding changes in the obstacles ahead. Therefore, the implementation of the MTATBOT’s autopilot mode mainly depends on the EWTBOT’s autopilot system.

The autopilot system equipped on the EWTBOT adopts a four-level functional architecture, including a sensing layer, a perception layer, a decision layer, and an execution layer. The sensing layer mainly consists of several kinds of sensors, such as Lidar, radar, RGB-D cameras, and IMU, providing information on obstacle distribution and robot posture parameters. The perception layer calculates and fuses the collected information to estimate the robot’s location and obstacle distance. The decision layer determines the obstacle avoidance strategy and calculates the certain future period trajectory of the robot based on the above information. The execution layer is in charge of controlling the robot to travel along the local planning path provided by the decision layer.

### 4.2. Environment Perception System of the EWTBOT

**Figure 7:**
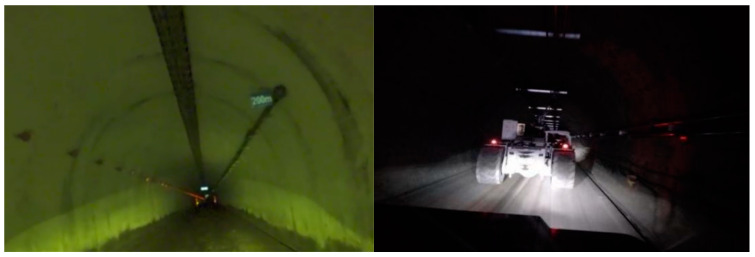
Perceptually degraded environment of an auxiliary transport roadway in the China Energy Group Bulianta Coal Mine.

Accurate and prompt environmental perception is crucial for the safe and autonomous operation of the EWTBOT in underground coal mines. Regrettably, the auxiliary transportation roadway environment where the EWTBOT operates often suffers from perceptual degradation, characterized by low or zero illumination, visually and geometrically similar surroundings, and occasional obscurants such as fog and dust, as illustrated in Figure 7. Meanwhile, considering the large scale of underground coal mine auxiliary transportation roadways (some of the roadways are dozens of kilometers long), these conditions make it difficult to achieve the desired perception results through traditional methods. Furthermore, due to the explosion-proof requirement of underground coal mines, commonly used detection sensors, such as Lidar and cameras, cannot be directly applied in the EWTBOT.

**Figure 8:**
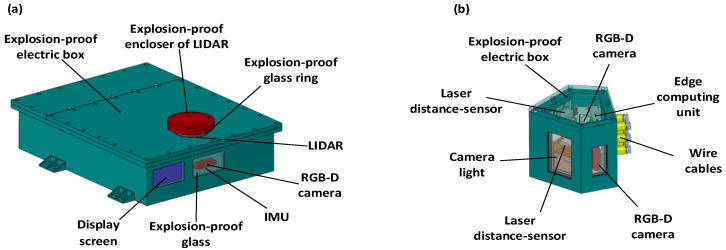
The sensors’ arrangement in the environment perception system in explosion-proof electric boxes. (**a**) The main explosion-proof electric box; (**b**) the corner explosion-proof electric box.

The environment perception system of the EWTBOT is specially designed for the conditions of underground transportation roadways. In order to obtain good perception performance in underground, perceptually degraded roadways, a combination of multi-type sensors, including Lidar, RGB-D cameras, radar, and IMU, is applied in the EWTBOT’s environment perception system. In addition, all of the sensors are explosion-proof and specially designed for the underground coal mine environment. As shown in Figure 8, all the optical sensors have been enclosed in explosion-proof electric boxes on the EWTBOT. With the help of specially designed explosion-proof glasses, the sensors can work normally in underground roadways, where the atmosphere is wet, dusty, and prone to explosions.

**Figure 9:**
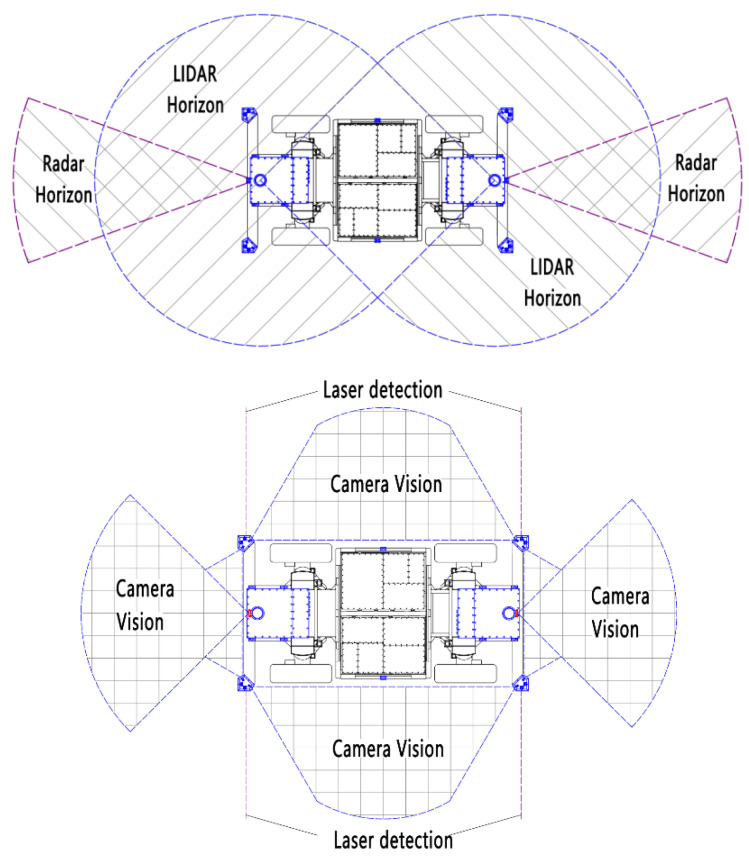
Detection range of the EWTBOT’s environment perception system. Top: Bird’s-eye view of the Lidar and millimeter-wave radar horizon on the EWTBOT. Bottom: Bird’s-eye view of RGB-D cameras and laser distance sensors’ horizon on the EWTBOT.

The arrangement and detection range of perception sensors applied on the EWTBOT are depicted in Figure 9. The EWTBOT is equipped with two Lidar sensors at the front and rear, each with a field of detection horizon of 270 degrees, thus achieving full coverage of the robot’s surrounding environment. Meanwhile, two-millimeter wave radars are installed separately in the front and rear of the EWTBOT, mainly for the detection and tracking of moving objects in the forward direction of the robot. Additionally, a total of 10 RGB-D cameras are arranged around the vehicle to achieve video coverage in the main directions of the robot. Additionally, several laser distance sensors are arranged on both sides of the robot, which can quickly obtain real-time information on the distance between the body and both sidewalls of the roadways, using the robot’s lateral position in the tunnel for judgment.

### 4.3. Localization Strategy of the EWTBOT

Accurate estimation of self-localization in underground coal mine transport roadways is another challenge for the autopilot system of the EWTBOT. Because GNSS-based localization is not applicable in subterranean environments, simultaneous localization and mapping based on the information collected by the environment perception system is essential for robot localization. However, the perceptual degradation of underground roadway environments is typically challenging for both Lidar-based and visual-based SLAM. In order to increase the accuracy of localization, our approach is to use a Lidar-centric SLAM solution fused by visual, IMU, and wheel rotation information, which can lead to more accurate and robust robot odometry. Moreover, in order to mitigate the inevitable accumulation of global drift over large-scale underground roadways when using SLAM to position the robot, auxiliary localization methods are simultaneously introduced into the strategy.

**Figure 10:**
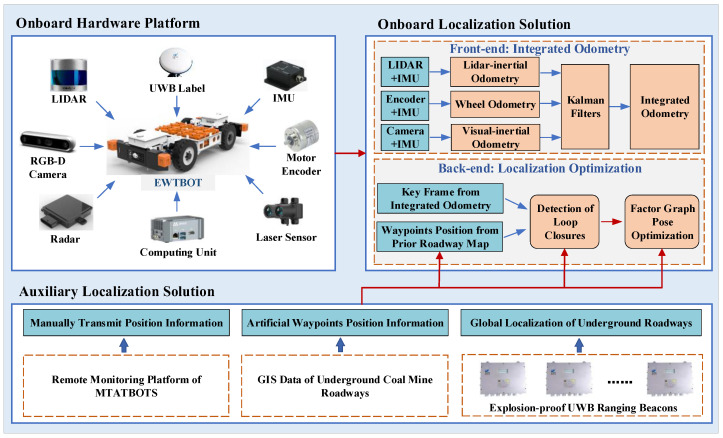
Architectural overview of the EWTBOT’s localization strategy.

It is important to note that when the robot travels in underground coal mines, its driving range is strictly limited between the sidewalls of the roadway. Compared with the length along the roadway direction, the roadway width (generally no more than 6 m) is almost negligible. Therefore, the localization of the EWTBOT mainly depends on its accurate odometry along the direction of the subterranean roadway. For the above reason, our localization strategy is to use multi-modal information to obtain accurate robot odometry, and then utilize effective auxiliary positioning methods to correct and optimize the robot’s trajectory and posture, with the aim of obtaining accurate vehicle position coordinates and heading angles to conduct the robot’s automatic motion control. Figure 10 shows the overview of the EWTBOT’s localization system architecture. As shown in the diagram, the EWTBOT’s localization system mainly consists of two parts: an onboard localization system and an auxiliary localization system.

(1) Onboard Localization Solution

Depending on the EWTBOT’s onboard hardware platform, the onboard localization solution is implemented through a complementary multi-modal SLAM system that includes two main components: the front-end and back-end components.

Front-end: Integrated Odometry. The front-end component of the onboard SLAM system abstracts data from the sensor into robot odometry. With the different types of data collected by relevant sensors, three components of the robot’s odometry information can be obtained separately, including Lidar-inertial odometry, wheel odometry, and visual-inertial odometry. The Lidar-inertial odometry component can fuse the Lidar point cloud matching results with the IMU data to derive the robot position and trajectory [14,15]. Our solution is a furtherance of an existing open-source implementation, LIO-SAM [16]. This tightly coupled Lidar inertial odometry framework can achieve accurate, real-time mobile robot trajectory estimation and map-building. In addition, the framework is suitable for multi-sensor fusion by formulating odometry atop a factor graph; thus, additional sensor measurements, such as wheel odometry and UWB position information, can be incorporated into the framework as new factors to eliminate sensor drift, which has significant importance, especially in a large-scale underground roadway environment [17]. The visual-inertial odometry component fuses the matching result of sparse ORB features [18] between consecutive images filmed by the RGB-D camera and the IMU data to estimate the robot odometry while reconstructing the environment [19,20]. Our solution builds upon the existing open-source implementation ORB-SLAM3 [21], which is a state-of-the-art visual odometry framework that can perform visual SLAM with an RGB-D camera. However, considering that visual odometry is not reliable in the low-texture underground roadway environment and is susceptible to illumination, the visual odometry component plays an auxiliary and complementary role in our strategy. The wheel odometry component can figure out the robot’s driving range based on the robot’s speed and heading angle, which are gathered by the motor encoder and IMU.

However, due to the perceptually degraded subterranean environment and poor road surface conditions, each kind of odometry information can be biased or even missing when the robot is traveling along the roadway. For example, long-range, corridor-like subterranean roadways and intersections that are structurally similar cause Lidar-based odometry to drift; uneven and slippery roadway surfaces lead to inaccurate wheel odometry; and drastic changes in illumination, poor illumination, dust, water puddles, and non-Lambertian surfaces render visual odometry unreliable [22]. Above all, it is hard to obtain accurate and robust localization results based on single-source odometry information. Therefore, a Kalman Filter is introduced into our strategy to consider all three odometry components simultaneously, as well as the integrated odometry information of the robot, which is more reliable and robust and can be figured out accordingly.

By dynamically adjusting the confidence levels of visual-inertial odometry, Lidar-inertial odometry, and wheel odometry during the Kalman fusion process according to the varying environmental conditions of the tunnels, the system’s robustness can be significantly enhanced, leading to more accurate results. At the current stage, the confidence levels of the three types of odometry are preset according to the specific mining environment where the robot will operate. In the future, the goal is to employ reinforcement learning and deep learning to enable real-time dynamic adjustments of these confidence levels across different mining environments.

Back-end: Localization Optimization. The back-end component of the onboard SLAM system is in charge of optimizing the robot’s trajectory and global map estimates by fusing the key frame information of the integrated odometry from the front-end component and the landmark position information from the a priori roadway map via a nonlinear estimator approach called pose graph optimization (PGO) [23]. The a priori map of underground roadways can be obtained from the geographic information system (GIS) of the coal mine or mapped from the onboard SLAM system. Position information regarding significant landmarks, such as underground traffic signs and signals, key intersections, and artificial markings, can be marked on the map as a priori localization markers. When the robot travels along the subterranean roadways, these markers can be captured by onboard RGB-D cameras with the help of YOLO [24]. YOLO produces a 2D bounding box around the detected marker, and then the position of the marker can be estimated according to the range of the bounding box center measured by the depth channel of the RGB-D camera.

**Figure 11:**
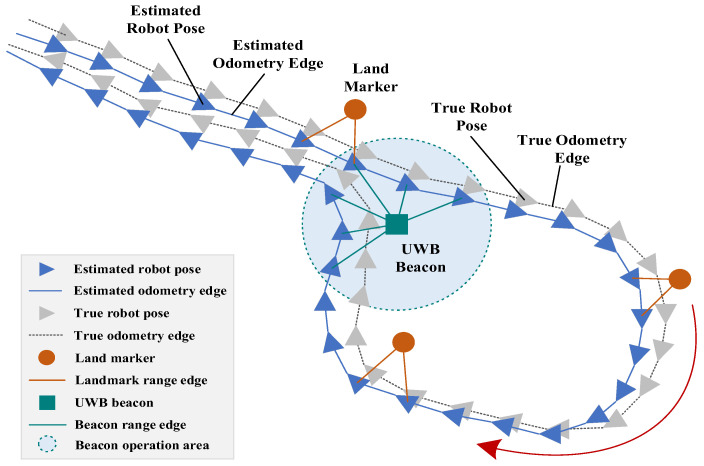
Factor graph and range measurements of the EWTBOT.

The pose graph of the EWTBOT is depicted in Figure 11. When the back-end component receives the front-end odometry information, a series of key frames are periodically instantiated in 1 m displacement intervals and the corresponding poses and odometry edges are added to the robot’s pose graph. When the back-end component receives the landmark measurements, a landmark point whose position can be obtained from the a priori roadway map is instantiated in the pose graph and an edge is added between the landmark and the corresponding observation pose. Based on the landmark’s position, the robot’s trajectory can be optimized accordingly and the spurious loop closure that occurs frequently in perceptually degraded subterranean roadways can be mitigated effectively. Therefore, the problem of the optimization of the robot’s odometry and trajectory can be formulated as pose graph optimization by integrating the a priori landmarks’ position constraints. This problem is implemented in our strategy based on the GTSAM [25] framework and uses the Levenberg–Marquardt algorithm to solve the nonlinear least squares problem.

(2) Auxiliary Localization Solution

To enhance the robustness and accuracy of EWTBOT localization, additional auxiliary positioning solutions should be incorporated alongside the existing subterranean roadway data. When landmark information for robot odometry optimization is insufficient, deploying radio beacons that transmit positional data to the robot proves to be a cost-effective and efficient strategy for localization optimization. This is due to their omnidirectional visibility, ease of deployment, and resilience to the perceptually degraded subterranean environment [8]. In our strategy, UWB devices are adopted as radio beacons to provide global positioning information for the EWTBOT.

Typically, positioning methods that use radio beacons require a dense distribution of beacons. However, considering that the deployment of enough radio beacons for large-scale underground roadways will significantly increase economic costs and that the effective radio range of each beacon is also severely limited by the geography of subterranean roadways, a limited number of explosive-proof UWB ranging beacons are sparsely deployed in the roadways, mainly in sharp turns and roadway intersections. These ranging beacons can provide global position information, enabling the robot to eliminate accumulated errors. As shown in Figure 11, when the robot travels into the UWB beacon operation area, a beacon range edge will be added to the pose graph between the beacon and the corresponding observation pose as a global constraint to correct the odometry and optimize the robot’s trajectory. In addition, all UWB beacons are uniquely identified so that the robot can quickly locate itself when passing by, effectively reducing the computational burden and avoiding spurious loop closure.

Furthermore, the remote monitor platform of the MTATBOT can also interact with the robot’s trajectory by manually transmitting position information to the robot in cases where the operator identifies a loop closure that has not been detected by the onboard SLAM system or when correcting spurious loop closures.

## 5. Motion Control of the EWTBOT

Driving stabilization and collision avoidance are two of the most crucial concerns when the EWTBOT operates in underground roadways, which have limited space and multiple traffic participants [26]. The motion control strategy of the robot adopts a hierarchical control architecture consisting of a decision-making layer and a motion execution layer. In the decision-making layer, a smooth and collision-free driving trajectory that meets the kinematic and dynamic constraints of the robot is calculated based on the changing environmental information. In the motion execution layer, the robot’s motion is decoupled into lateral and longitudinal motion under the Frenet coordinate system [27], with the planned driving path as the coordinate axis. The lateral motion controller manipulates the steering system according to the deviation between the robot’s actual position and the planned trajectory. The longitudinal controller controls the traction motor and the brake system to track the position and speed of the planned trajectory. In this section, the motion model of the EWTBOT is described based on dynamics analysis, and then the motion control scheme of the robot is presented.

### 5.1. Motion Forms of the EWTBOT

**Figure 12:**
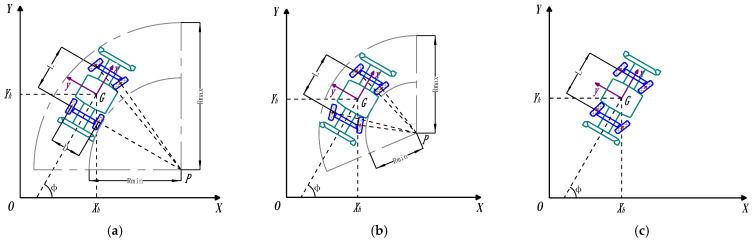
Multi-type motion forms of the explosion-proof wheeled transport robot. *G* is the robot CoG, positioned at *X_h_* and *Y_h_* in the global Cartesian coordinate system *XOY*. *xGy* is the robot body coordinate system with the robot’ longitudinal axis as the *x*-axis and its lateral direction as the *y*-axis. *L* is the robot’s axis distance. *D* is the robot’s wheel distance. *P* is the instantaneous center of steering of the robot. ϕ is the yaw angle of the robot. *R_min_* is the robot’s minimum turning radius. *R_max_* is the robot’s maximum turning radius. (**a**) Front/Rear-wheel steering. (**b**) Four-wheel steering. (**c**) Crab-walking.

The EWTBOT is equipped with two sets of Ackerman steering mechanisms at the front and rear, respectively, which can realize a variety of motion forms to improve its maneuverability. Three motion forms of the robot are shown in Figure 12, including front/rear-wheel steering, four-wheel steering, and crab-walking. Compared with front-wheel steering or rear-wheel steering, four-wheel steering can effectively reduce the turning radius of the robot. Crab-walking makes the robot travel diagonally, i.e., the driving direction is deflected by an angle between the longitudinal axis of the vehicle. In the crab-walking mode, the robot can easily perform obstacle avoidance maneuvers in subterranean roadways of limited space. Moreover, the two steering systems serve as a backup for each other. If one of the steering systems fails, the robot can still complete the steering maneuver.

### 5.2. Dynamic Model of the EWTBOT

**Figure 13:**
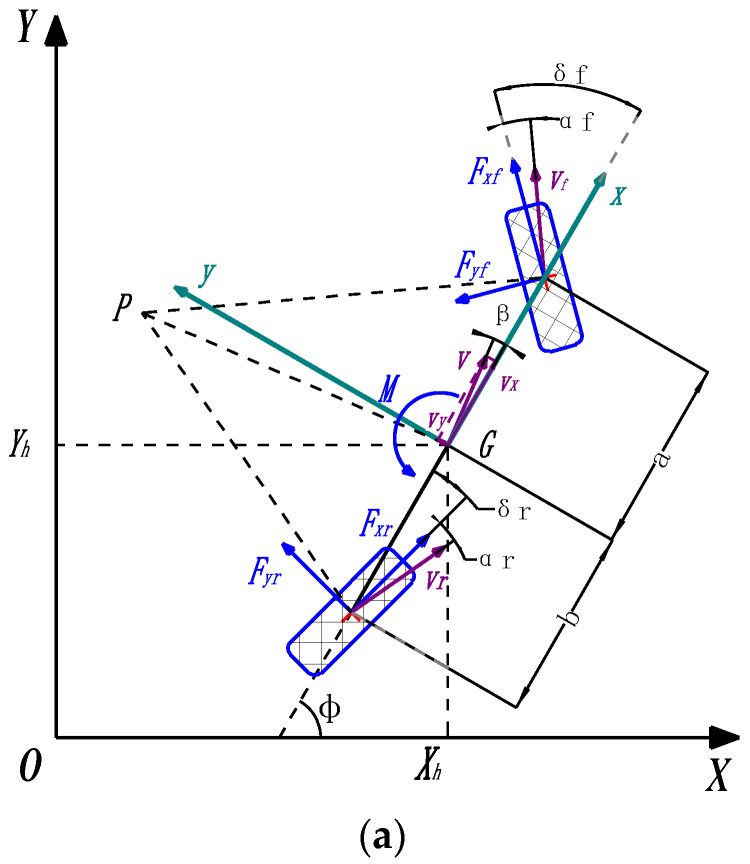

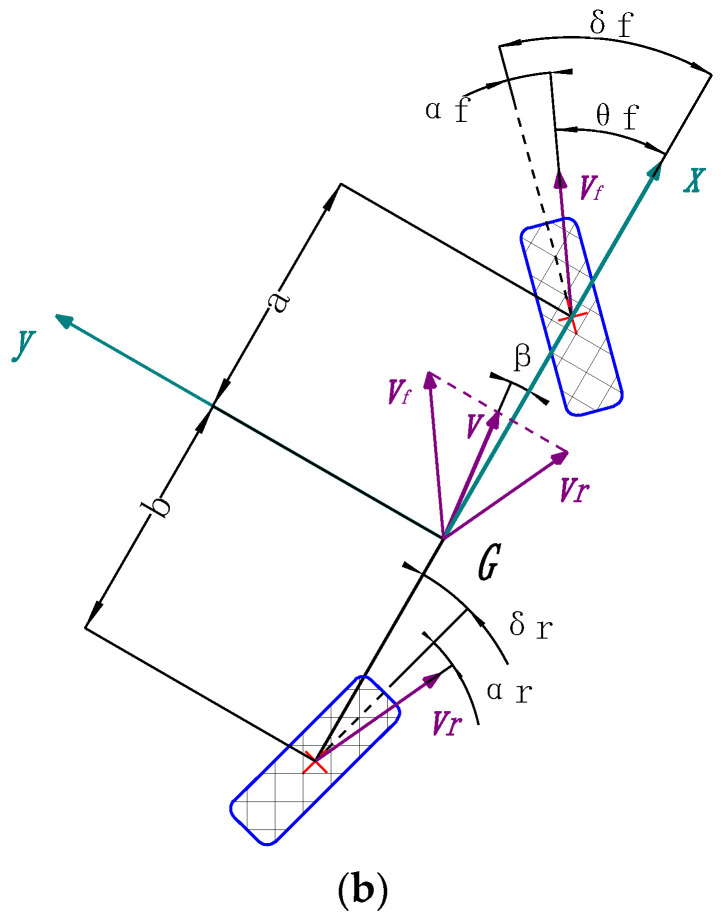
Two-degrees-of-freedom model of the explosion-proof wheeled transport robot. *G* is the robot’s CoG; *P* is the instantaneous center of steering of the robot; *F_yf_* and *F_yr_* are the lateral tire forces of the front and rear axles; *F_xf_* and *F_xr_* are the components of force provided by the front and rear tires, respectively, in their direction of rolling; *V* is the robot’s velocity vector in the center of gravity, which has a longitudinal component *v_x_* and a lateral component *v_y_*, which identify the sideslip angle *β*; *V_f_* is the front tire’s velocity vector; and *V_r_* is the rear tire’s velocity vector. (**a**) Dynamic analysis of the 2-DOF model. (**b**) Velocity vectors’ geometrical relationship in the model.

The effectiveness of the motion control of the EWTBOT should be based on a correct and reliable vehicle model [28,29]. A feasible local path planning trajectory generated by the decision-making layer needs to satisfy the kinematic and dynamic constraints of the robot [30,31]. In order to develop a reasonable control scheme and mitigate the computational burden, a planar 2-DoF bicycle model [32] is employed to represent the robot, as depicted in Figure 13a, which utilizes small angle assumptions and the approximation that the tires on each axle can be lumped together. *G* is the center of gravity of the robot, which is positioned at *X_h_* and *Y_h_* in the global Cartesian coordinate system *XOY*. ϕ is the robot’s yaw angle between the Earth’s coordinate system’s *X*-axis and the robot‘s longitudinal *x*-axis. Thus, the robot’s position and posture in the Earth’s fixed coordinate system can be defined by the vector $[\begin{array}{ccc} X_{h} & Y_{h} & \phi \end{array}{]}^{T}$. Assuming that the robot drives at a constant speed *v_x_* in the direction of the robot’s longitudinal *x*-axis, the bicycle model consisting of the lateral and yaw dynamics in the robot body coordinate system *xGy* and the force and torque equilibrium of the 2-DOF bicycle model in the lateral direction can be derived, as follows, according to geometric relationships and dynamic analysis:

(1) $$\sum F_{y}=m\left({\dot{v}}_{y}+v_{x}\cdot \dot{\phi }\right)\approx F_{yf}\cos\delta_{f}+F_{yr}\cos\delta_{r}\approx C_{\alpha f}\cdot \alpha_{f}+C_{\alpha r}\cdot \alpha_{r}$$

(2) $$\sum M=I_{z}\ddot{\phi }\approx a\cdot F_{yf}\cos\delta_{f}-b\cdot F_{yr}\cos\delta_{r}\approx a\cdot C_{\alpha f}\cdot \alpha_{f}-b\cdot C_{\alpha r}\cdot \alpha_{r}$$

where *F_y_* is the force of the model in the direction of the robot’s lateral *y*-axis; *M* is the turning torque about the *z*-axis; *m* is the robot’s mass; *I_z_* is the moment of inertia about the *z*-axis; ϕ is the yaw angle; *v_x_* and *v_y_* are the components of the velocity vector in the center of gravity along the *x*-axis and the lateral *y*-axis of the robot body coordinate system; and *β* is the sideslip angle of the robot in the center of gravity. *F_yf_* and *F_yr_* are the lateral tire forces, perpendicular to the rolling direction of the tire, and proportional to the slip angle, *α*, between the local velocity vector and its forward direction; *α_f_* and *α_r_*, respectively, denote the front and rear tire slip angles; *a* and *b* are the distances from the center of gravity of the vehicle to the front and rear axles; *δ_f_* and *δ_r_* are the steering angles of the front and rear wheels with respect to the robot, which are assumed to be small angles; and *C_αf_* and *C_αr_* are the tire stiffness of the front and the rear tire pairs, respectively.

Based on the velocity vectors’ geometric relationships with the robot’s center of gravity, the front and rear tires, depicted in Figure 13b, and the slip angles can be obtained in the following equations:

(3) $$\alpha_{f}=-\left(\delta_{f}-\theta_{f}\right)=\frac{\dot{\phi }\cdot a+v_{y}}{v_{x}}-\delta_{f}\approx \beta +\frac{\dot{\phi }\cdot a}{v_{x}}-\delta_{f}$$

(4) $$\alpha_{r}=-\left(\frac{\dot{\phi }\cdot b-v_{y}}{v_{x}}-\delta_{r}\right)\approx \beta +\delta_{r}-\frac{\dot{\phi }\cdot b}{v_{x}}$$

where $v_{y}/v_{x}=\tan\beta \approx \beta$.

Furthermore, in order to meet the needs of the robot, such as multiple motion forms, the control of the steering angles of the front and rear tires follows the following rule:

(5) $$\delta_{r}=\xi \cdot \delta_{f}$$

where ξ∈ [−1,1], ξ  is the control scale factor.

Next, by bringing Equations (3)–(5) into Equations (1) and (2), the parametric 2-DOF vehicle model and the expression for the lateral position of the robot can be written as follows:

(6) $$\left\{\begin{array}{c} \ddot{y}=\left(\frac{C_{\alpha f}+C_{\alpha r}}{mv_{x}}\right)\dot{y}+\left(\frac{C_{\alpha f}\cdot a-C_{\alpha r}\cdot b}{mv_{x}}-v_{x}\right)\dot{\phi }+\left(\frac{-C_{\alpha f}+\xi \cdot C_{\alpha r}}{m}\right)\delta_{f}\\ \ddot{\phi }=\left(\frac{a\cdot C_{\alpha f}-b\cdot C_{\alpha r}}{I_{z}v_{x}}\right)\dot{y}+\left(\frac{a^{2}\cdot C_{\alpha f}+b^{2}\cdot C_{\alpha r}}{I_{z}v_{x}}\right)\dot{\phi }+\left(\frac{-a\cdot C_{\alpha f}-\xi \cdot b\cdot C_{\alpha r}}{I_{z}}\right)\delta_{f} \end{array}\right.$$

where *y* is the robot’s lateral displacement; ϕ is the yaw angle; and *δ_f_* is the steering angle of the front wheel. By defining $X=[\begin{array}{cc} \dot{y} & \dot{\phi } \end{array}{]}^{T}$ as the state vector and $U=[\delta_{f}]$ as the control vector, the robot model described in Equation (6) can be converted into the state equation as:

(7) $$\dot{X}=AX+BU$$

where

$$A=\left[\begin{array}{cc} \frac{C_{\alpha f}+C_{\alpha r}}{mv_{x}} & \frac{C_{\alpha f}\cdot a-C_{\alpha r}\cdot b}{mv_{x}}-v_{x}\\ \frac{a\cdot C_{\alpha f}-b\cdot C_{\alpha r}}{I_{z}v_{x}} & \frac{a^{2}\cdot C_{\alpha f}+b^{2}\cdot C_{\alpha r}}{I_{z}v_{x}} \end{array}\right],\;B=\left[\begin{array}{c} \frac{-C_{\alpha f}+\xi \cdot C_{\alpha r}}{m}\\ \frac{-a\cdot C_{\alpha f}-\xi \cdot b\cdot C_{\alpha r}}{I_{z}} \end{array}\right]$$

### 5.3. Path-Following Error Dynamic Model

**Figure 14:**
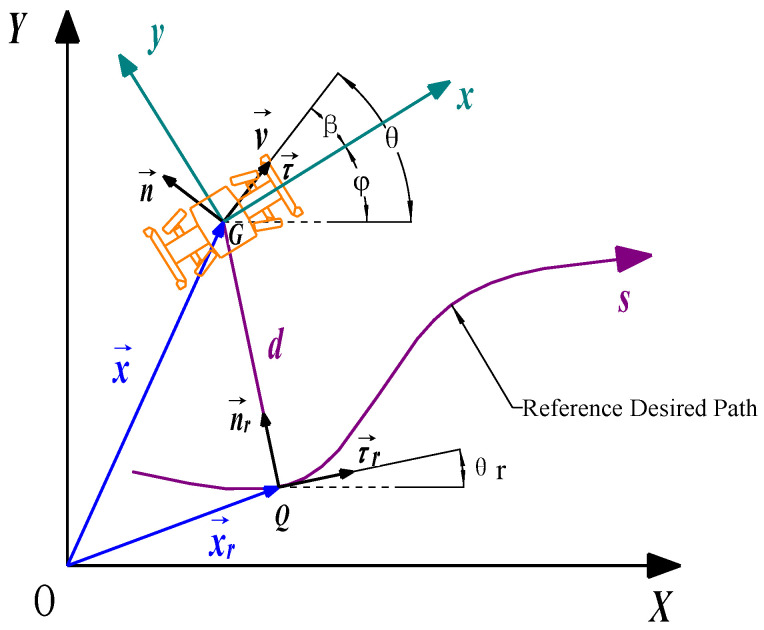
Path-following error dynamics model of the explosion-proof wheeled transport robot. $\vec{v}$ is the robot’s actual velocity vector; *G* is the robot’s CoG; *Q* is the orthogonal projection of *G* on the desired path; and *d* is the distance from *G* to *Q*.

The control objective of the EWTBOT is to ensure that the position and the heading angle of the robot track the planned reference path. Using the desired path as the reference line, the robot’s movement can be decoupled separately as lateral and longitudinal motions based on the Frenet coordinate system. In the coordinate system, the direction along the desired path is taken as the longitudinal axis, and the direction perpendicular to the reference path is taken as the lateral axis [33]. As shown in Figure 14, the reference desired path of the EWTBOT, on which the robot is supposed to drive, is depicted as the curve *s*. In addition, *d* represents the distance from the center of gravity of the robot to the closest point *Q* on the desired path, i.e., the orthogonal projection of *G* on the reference path. The robot’s position under the Frenet coordinate system can be depicted as the longitudinal displacement *s* and the lateral displacement *d*. Thus, $\dot{s}$ is the robot’s desired velocity at its projection point *Q*, along the tangential direction of the desired path.

Under the global Cartesian coordinate system *XOY*, the actual position and the desired position on the reference path of the robot can be represented separately in position vectors of $\vec{x}$ and ${\vec{x}}_{r}$. θ and $\theta_{r}$, respectively, represent the robot’s actual heading angle and the desired heading angle on the reference trajectory. Then, defining *d* as the lateral position error and $\left(\theta -\theta_{r}\right)$ as the heading error of the robot, the objective of the robot’s lateral control is to globally and asymptotically minimize the two kinds of path-following errors. In addition, defining $\left(\left|\vec{v}\right|-\left|\dot{s}\right|\right)$ as the robot’s velocity magnitude error, the objective of the robot’s longitudinal control is to minimize the longitudinal position error and the velocity error. In addition, assuming that $\vec{\tau },\vec{n}$ are orthogonal unit vectors at point *G*, ${\vec{\tau }}_{r},{\vec{n}}_{r}$ are orthogonal unit vectors at point *Q*, and the directions of $\vec{n}$ and ${\vec{n}}_{r}$ are consistent with the vectors of the actual velocity and the desired velocity of the robot, the lateral displacement *d* and the lateral velocity $\dot{d}$ can be attained as follows:

(8) $${\vec{x}}_{r}+d{\vec{n}}_{r}=\vec{x}\Rightarrow d=\left(\vec{x}-{\vec{x}}_{r}\right)\cdot {\vec{n}}_{r}$$

(9) $$\dot{d}=\left(\dot{\vec{x}}-{\dot{\vec{x}}}_{r}\right)\cdot {\vec{n}}_{r}+\left(\vec{x}-{\vec{x}}_{r}\right)\cdot {\dot{\vec{n}}}_{r}=\left|\vec{v}\right|\cos\left\langle \vec{\tau },{\vec{n}}_{r}\right\rangle =\left|\vec{v}\right|\sin\left(\theta -\theta_{r}\right)$$

where $\dot{\vec{x}}=\left|\vec{v}\right|\vec{\tau }$; ${\dot{\vec{x}}}_{r}=\dot{s}{\vec{\tau }}_{r}$; ${\dot{\vec{n}}}_{r}=-\kappa {\vec{\tau }}_{r}\dot{s}$; κ represents the curvature of the desired path.

The robot’s desired velocity can be calculated in the following equation:

(10) $$\dot{s}=\frac{\left|\vec{v}\right|\cos\left(\theta -\theta_{r}\right)}{1-\kappa \cdot d}$$

Considering that θ=ϕ+β and $v_{x}=\left|\vec{v}\right|\cos\beta ,\;v_{y}=\left|\vec{v}\right|\sin\beta$, Equations (9) and (10) can be written as follows:

(11) $$\left\{\begin{array}{c} \dot{d}=v_{y}cos\left(\phi -\theta_{r}\right)+v_{x}\sin\left(\phi -\theta_{r}\right)\approx v_{y}+v_{x}\left(\phi -\theta_{r}\right)\\ \dot{s}=\frac{v_{x}\cos\left(\phi -\theta_{r}\right)-v_{y}\sin\left(\phi -\theta_{r}\right)}{1-\kappa \cdot d}\approx \frac{v_{x}-v_{y}\left(\phi -\theta_{r}\right)}{1-\kappa \cdot d} \end{array}\right.$$

where $\left(\phi -\theta_{r}\right)$ is assumed to be a small angle.

Defining that $e_{d}=d$ is the lateral error and $e_{\phi }=\phi -\theta_{r}$ is the heading error, the following equations can be attained:

(12) $$\left\{\begin{array}{c} v_{y}={\dot{e}}_{d}-v_{x}e_{\phi }\\ {\dot{v}}_{y}={\ddot{e}}_{d}-v_{x}{\dot{e}}_{\phi }\\ \dot{\phi }={\dot{e}}_{\phi }+{\dot{\theta }}_{r}\\ \ddot{\phi }={\ddot{e}}_{\phi }+{\ddot{\theta }}_{r}\approx {\ddot{e}}_{\phi } \end{array}\right.$$

Next, by bringing Equation (12) into Equation (6), the path-following error dynamics model of the EWTBOT can be written as follows:

(13) $$\left\{\begin{array}{c} {\ddot{e}}_{d}-v_{x}{\dot{e}}_{\phi }=\left(\frac{C_{\alpha f}+C_{\alpha r}}{mv_{x}}\right)\left({\dot{e}}_{d}-v_{x}e_{\phi }\right)+\left(\frac{C_{\alpha f}\cdot a-C_{\alpha r}\cdot b}{mv_{x}}-v_{x}\right)\left({\dot{e}}_{\phi }+{\dot{\theta }}_{r}\right)+\left(\frac{-C_{\alpha f}+\xi \cdot C_{\alpha r}}{m}\right)\delta_{f}\\ {\ddot{e}}_{\phi }=\left(\frac{a\cdot C_{\alpha f}-b\cdot C_{\alpha r}}{I_{z}v_{x}}\right)\left({\dot{e}}_{d}-v_{x}e_{\phi }\right)+\left(\frac{a^{2}\cdot C_{\alpha f}+b^{2}\cdot C_{\alpha r}}{I_{z}v_{x}}\right)\left({\dot{e}}_{\phi }+{\dot{\theta }}_{r}\right)+\left(\frac{-a\cdot C_{\alpha f}-\xi \cdot b\cdot C_{\alpha r}}{I_{z}}\right)\delta_{f} \end{array}\right.$$

By defining $E_{rr}={\left[\begin{array}{cccc} e_{d} & {\dot{e}}_{d} & e_{\phi } & {\dot{e}}_{\phi } \end{array}\right]}^{T}$ as the state vector, the path-following error dynamics model described in Equation (13) can be converted into the state equation as:

(14) $${\dot{E}}_{rr}=\tilde{A}E_{rr}+\tilde{B}U+\tilde{C}{\dot{\theta }}_{r}$$

where

$$\tilde{A}=\left(\begin{array}{cccc} 0 & 1 & 0 & 0\\ 0 & \frac{C_{f}+C_{r}}{mv_{x}} & -\frac{C_{f}+C_{r}}{m} & \frac{aC_{f}-bC_{r}}{mv_{x}}\\ 0 & 0 & 0 & 1\\ 0 & \frac{aC_{f}-bC_{r}}{I_{z}v_{x}} & -\frac{aC_{f}-bC_{r}}{I_{z}} & \frac{a^{2}C_{f}+b^{2}C_{r}}{I_{z}v_{x}} \end{array}\right),\;\tilde{B}=\left(\begin{array}{c} 0\\ \frac{-C_{f}+\xi C_{r}}{m}\\ 0\\ \frac{-\xi bC_{r}-aC_{f}}{I_{z}} \end{array}\right),\;\tilde{C}=\left(\begin{array}{c} 0\\ \frac{aC_{f}-bC_{r}}{mv_{x}}-v_{x}\\ 0\\ \frac{a^{2}C_{f}+b^{2}C_{r}}{I_{z}v_{x}} \end{array}\right)$$

Furthermore, by using Euler’s approximation, Equation (14) can be discretized at sample time *t_s_*, and the discrete-time state equation can be obtained as:

(15) $$E_{rr}\left(k+1\right)={\tilde{A}}_{d}E_{rr}\left(k\right)+{\tilde{B}}_{d}U\left(k\right)+{\tilde{C}}_{d}{\dot{\theta }}_{r}\left(k\right)$$

where ${\tilde{A}}_{d}={\left(I-\frac{\tilde{A}t_{s}}{2}\right)}^{-1}\left(I+\frac{\tilde{A}t_{s}}{2}\right)$, ${\tilde{B}}_{d}={\left(I-\frac{\tilde{A}t_{s}}{2}\right)}^{-1}\tilde{B}t_{s}$, ${\tilde{C}}_{d}={\left(I-\frac{\tilde{A}t_{s}}{2}\right)}^{-1}\tilde{C}t_{s}$, and *I* denote the identity matrix.

### 5.4. Motion Control Scheme

**Figure 15:**
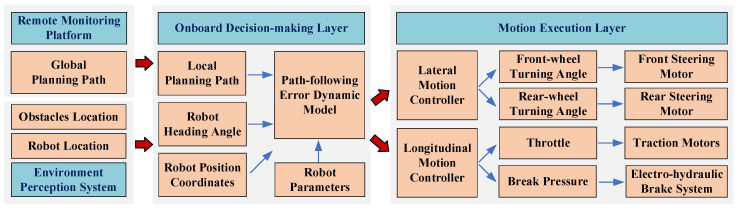
Motion control scheme framework of the MTATBOT.

Depending on the path-following error dynamics model, the control strategy of the EWTBOT is developed as shown in Figure 15. When the EWTBOT receives the global planning path from the remote monitoring platform, the onboard decision-making layer of the motion control system will generate a collision-free local planning path based on the global path. The local planning path is generated according to the distribution information of surrounding obstacles provided by the robot’s environment perception system. The path consists of a series of adjacent discrete trajectory points, each of which contains the desired position coordinates and heading angle of the robot in certain planning moments, along with information on the robot’s desired velocity and acceleration. Meanwhile, the current position and the heading angle of the robot can be acquired through the robot localization system. The above information, as well as the robot’s attribute parameters, will be passed into the path-following error dynamic model of the EWTBOT as input data.

The motion execution layer is responsible for manipulating the explosion-proof wire-controlled system to walk along the local planning path. In our strategy, the robot motion is decoupled into the lateral and longitudinal motion under the Frenet coordinate frame. The lateral motion control of the robot can be figured out by minimizing the errors of the lateral displacement and the heading angle between the robot’s current state and the reference trajectory point. The robot’s lateral control outputs are transmitted to the steering motors, which manage the turning angles of both the front and rear wheels. On the other hand, the longitudinal motion controller is in charge of tracking the desired velocity and acceleration of the reference trajectory point based on the robot’s actual speed and acceleration. Therefore, the robot’s longitudinal control outputs are conveyed as throttle and brake pressure to the traction motors and the electro-hydraulic brake system, respectively.

## 6. Experiment and Results

### 6.1. Simulation Testing

**Figure 16:**
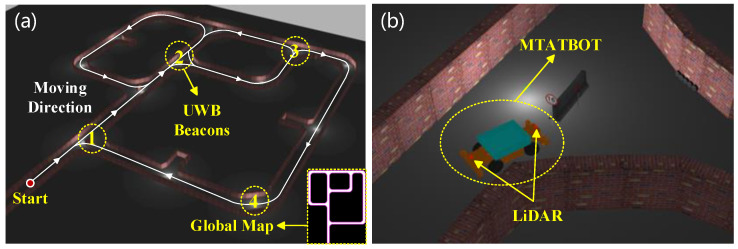
Simulation testing model of the MTATBOT. (**a**) Simulated underground roadway environment. A solid white line represents the traverse through the roadway, while arrows depict the direction of the traverse; yellow circles denote the action area of UWB beacons. (**b**) Simulated model of the MTATBOT.

A simulated model of the MTATBOT based on ROS and a virtual scenario of underground coal mine auxiliary transport roadways are established in Gazebo to facilitate the simulation testing of the robot, as shown in Figure 16. The virtual model of the MTATBOT was built according to its real size and system configuration, which is equipped with a simulated environment perception system and a motion control system. Under the control of the path-following algorithm based on the proposed motion control strategy, the virtual robot can travel along the desired trajectory in the simulated underground roadways. Meanwhile, with the help of the SLAM algorithm, the environment perception system can estimate the motion state and position of the robot based on the acquired environment information and onboard sensor data. Accordingly, the robot’s trajectory and the roadway map can be obtained simultaneously. In order to test the effectiveness of localization and the control strategy of the MTATBOT in the virtual underground scene, several kinds of SLAM solutions are implemented, relying on the simulation model to estimate the robot’s odometry and to build the scenario map. The algorithms are tested separately by running the same traveling route; therefore, the robot’s odometry was figured out through different localization solutions and can be compared on the basis of the actual robot’s trajectory.

**Figure 17:**
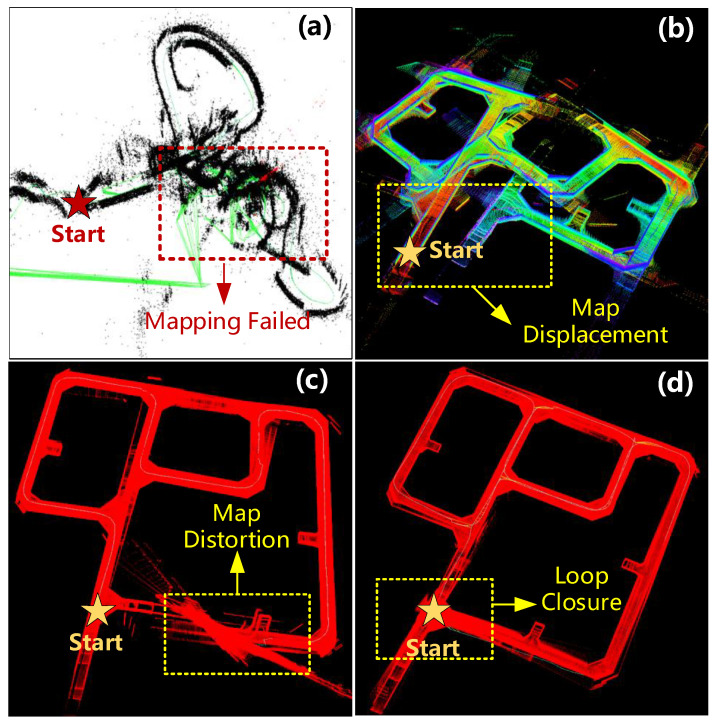
Simulation tests of the MTATBOT’s simultaneous localization and mapping solutions. (**a**) The results of Visual-based SLAM; (**b**) the results of Lidar-based SLAM; (**c**) the results of Lidar-inertial-based SLAM; and (**d**) the results of integrated-odometry-based SLAM.

Figure 17 shows the mapping results of the virtual underground roadways obtained through four different SLAM solutions. In the simulated underground roadway scenario, the vision-based SLAM can hardly execute the localization and mapping task independently, as shown in Figure 17a, because of the featureless environment and varying illumination. As for the Lidar-based SLAM, it significantly underestimates the robot motion in long symmetric corridor-like roadway scenarios because of the lack of detectable geometric features. In addition, intersections with similar structures can easily cause spurious loop closures. The simulated scenario mapping results with Lidar-based SLAM are shown in Figure 17b. With the fusion of IMU data and the wheel odometry information of the robot, the Lidar-inertial-based SLAM can estimate the robot’s odometry more accurately and obtain better mapping results, as shown in Figure 17c. However, as it is subject to cumulative errors, the robot’s odometry obtained by the algorithm inevitably drifts to a certain extent after a long driving route and several sharp turns, which leads to deviations in the mapping result.

Finally, in the virtual underground scenario, the proposed integrated-odometry-based SLAM algorithm of this paper is tested and proven to be effective in eliminating cumulative errors and obtaining accurate mapping results, as shown in Figure 17d. With the help of a priori localization vision markers and sparse global position signals provided by virtual UWB beacons installed in roadway intersections, more accurate and robust robot positions and odometry can be acquired by combining Lidar-inertial odometry, visual-inertial odometry, and wheel odometry.

**Figure 18:**
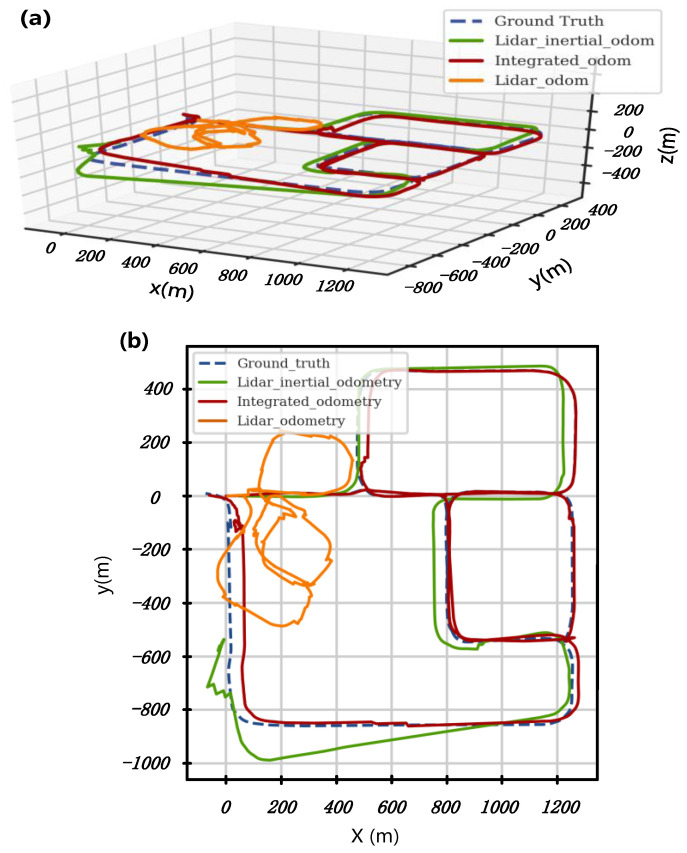
Comparison of MTATBOT trajectories based on different odometry information with the Ground Truth. (**a**) The comparative results on a three-dimensional scale; (**b**) the comparative results on a planar scale.

Furthermore, the robot odometry obtained by different algorithms is put into the same coordinate system and compared with the ground truth of the robot trajectory, as shown in Figure 18. The results show that the proposed integrated-odometry-based localization solution and motion control strategy of the robot are feasible and effective in the simulated scenario of underground coal mine auxiliary transport roadways. Table 2 illustrates the performance evaluation of these methods using the root mean square error (RMSE) of both absolute pose error (APE) and relative pose error (RPE), benchmarked against the ground truth robot pose from Gazebo. Compared to other algorithms, the proposed integrated-odometry-based SLAM algorithm achieves superior performance in both state estimation accuracy and roadway mapping quality during simulation testing. The RMSE of APE and RPE relative to the ground truth are 2.53 m and 0.151 m, respectively, the lowest among the tested methods. As shown in Figure 18, with the assistance of the data from the wheel encoder and the positional information of UWB beacons, the robot’s trajectory as estimated by our method aligns most closely with the ground truth.

### 6.2. Field Experiments in an Underground Coal Mine

**Figure 19:**
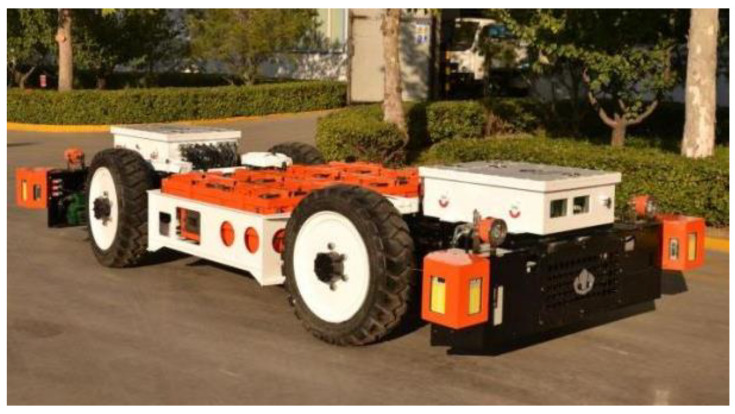
Physical prototype of the MTATBOT.

To validate the practicality of our robot system, field experiments were conducted in an active underground coal mine, the Cunchao Tower Coal Mine, located in northwest China. The field experiments were conducted using the prototype of the MTATBOT, as shown in Figure 19. The experiments were divided into two phases. First, the MTATBOT robot started from the starting point and traveled along the tunnel for a round trip back to the starting point, while simultaneously creating a map of the roadway environment it has traveled through, establishing a roadway map of the test coal mine. Second, we conducted quantitative experiments by comparing our SLAM method against other algorithms in the test underground roadways, using total station measurements of pre-installed targets as the ground truth. We also evaluated whether the robot could return to the starting point with minimal accumulative drifts.

The field experiment involved a quantitative analysis of our proposed method by comparing it with other representative algorithms. Obtaining the ground truth for the robot’s trajectory and the roadway map in large-scale underground coal mine environments is a challenging task. To evaluate our algorithm, we adopted the method of measuring start-to-end drift, wherein the robot starts and stops at the same location within the tunnel. Meanwhile, to obtain a relatively accurate ground truth of position in large-scale subterranean laneways of the test coal mine, we pre-installed a series of spherical targets along the test tunnel.

**Figure 20:**
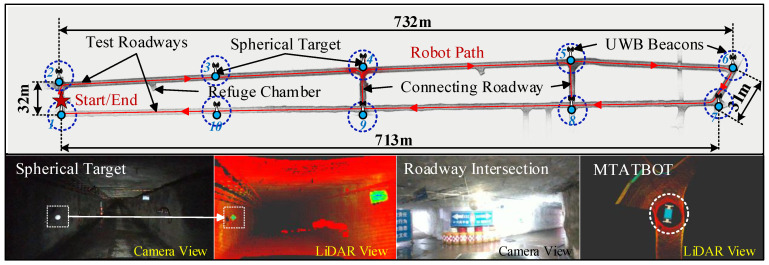
Mapping result of test underground roadways based on the MTATBOT. A total of 10 spherical targets were pre-arranged along the roadways, and UWB beacons were also deployed with each target to provide location information. The MTATBOT embarked on its journey from the starting point, traversed the length of the tunnel, and returned to its origin, successfully conducting environmental detection and mapping.

The spherical targets, depicted in Figure 20, are made of high-reflectivity materials. Owing to their uncommon spherical shape and high surface reflectivity in the underground coal mine setting, these targets can be readily differentiated from the tunnel background when exposed to LiDAR illumination. Each target has a precisely known diameter of 300 mm. The spatial locations of these spherical targets were measured by an electronic total station. These spherical targets with known positions were pre-installed in the underground roadways at locations such as intersections, corners, and merge points, to serve as the positional ground truth for the experiment.

**Figure 21:**
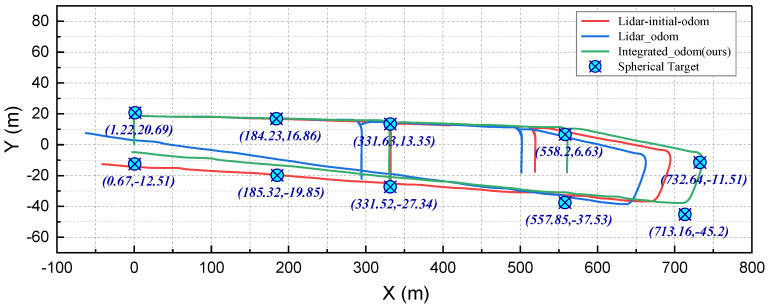
The robot’s traversed trajectories obtained by the used methods during the field experiment. The spherical targets’ positions relative to the start point, which were measured by an electronic total station, are also labeled in the graph. Compared to the other algorithms with large start-to-end drifts, the proposed method can obtain a closed traversed path when the robot returned to the start point.

As depicted in Figure 20, the underground experiment field comprises two parallel long tunnels linked by several connecting roadways. Ten spherical targets are dispersed throughout the roadways, primarily installed at the intersections of the tunnels. A series of UWB beacons were also deployed with each target to provide location information. The positions of these spherical targets relative to the start point were measured using an electronic total station. The positions of these targets are also used as ground truth positioning points for the experiment, as shown in Figure 21.

The robot moved from the start point along the route marked by the red solid line in Figure 20 and eventually returned to the start point, forming a closed loop of the traversed path. The robot’s traversed paths obtained using our method and other representative algorithms in the underground experiments are depicted in Figure 21. Since there is no available ground truth for the robot’s trajectory, we quantitatively compared the algorithms based on the start-to-end translation errors. As shown in Table 3, the total start-to-end translation error of the robot path obtained with the proposed method is 7.43 m, which is significantly less than the other algorithms.

## 7. Conclusions and Future Work

In this work, a new type of transport robot system, MTATBOT, is introduced for intelligent auxiliary transportation in underground coal mines, aimed at executing automated and unmanned subterranean transport tasks. This robot system is specially designed for explosion-proof, long-range, and perception-degraded underground roadway scenarios. The deployment and promotion of the MTATBOT can significantly decrease the number of workers required in underground coal mines by facilitating automated and unmanned material transportation. This advancement not only enhances safety by effectively preventing underground transportation accidents but also offers substantial economic and social benefits. It is estimated that roughly 50 MTATBOTs can fulfill the transportation needs of an underground coal mine with an annual production capacity of ten million tons. This implementation can reduce the number of underground personnel by 25% and decrease the incidence of related accidents by over 50%, resulting in annual savings of more than CNY five million in personnel and other related costs for the coal mine.

This paper describes the structural composition of the robot system and the functional implementation of the robot autopilot system. To solve the perception and localization challenges of GNSS-denied subterranean roadway environments, the integrated-odometry-based SLAM solution is proposed and applied in the robot localization strategy. The proposed algorithm can achieve accurate and robust robot odometry and mapping results by considering Lidar-inertial odometry, visual-inertial odometry, and wheel odometry simultaneously. In order to mitigate the influence of accumulative errors from onboard sensors, the global position information provided by UWB beacons sparsely arranged in underground roadways is also taken into account in the localization algorithm. Furthermore, the robot path-following motion control strategy is presented based on the dynamic model of the robot. Finally, in order to validate the proposed localization algorithm and motion control strategy, the robot virtual model and the simulated underground coal mine auxiliary transport roadway scenario are established. The simulation testing and field experiment results indicate that the proposed localization solution and control strategy of the robot are suitable for underground coal mines.

Given the complexity of underground transport roadway conditions, accurately replicating the robot system’s operating environment in different coal mines within the current test scenario is still a challenge. In the future, it is crucial to further test and adapt the proposed algorithms and control strategies across various subterranean coal mine environments. Additionally, while the current robot control strategy primarily concentrates on tracking the planned route, there is a pressing need to enhance and optimize autonomous collision avoidance maneuvers to ensure safe and smooth operation in the narrow, space-constrained underground roadways.

## Figures and Tables

**Table 1:** Technical specifications of the Explosion-proof Wheeled Transport Robot.

Items	Parameters
Robot Mass	5000 kg
Maximum Load Capacity	5000 kg
Robot Body Size	4500 × 2000 × 1000 mm
Maximum Speed	40 km/h
Climbing Capacity	14°
Turning Radius	≤5400 (outer)/≥2800 (inner) mm
Maximum Driving Range	80 km
Battery Capacity	64 kWh
Installed Power	2 × 46 kW
Perception Range	360°
Perception Distance	≥20 m

**Table 2:** RMSE Translation Error with regards to the Ground Truth (meters) (Best in Bold).

Methods	Lidar Odometry	Lidar-Inertial Odometry	Integrated Odometry (Ours)
APE	97.62	15.35	**2.53**
RPE	0.685	0.283	**0.151**

**Table 3:** Start-to-end Translation Errors (meters) (Best in Bold).

Errors	Lidar Odometry	Lidar-Inertial Odometry	Integrated Odometry (Ours)
X-axis	69.42	45.55	**4.2**
Y-axis	8.12	14.42	**3.23**
Total	77.54	59.97	**7.43**

## Data Availability

Dataset available on request from the authors.
